# 

**Published:** 1893-05

**Authors:** 


					﻿Entered at the Post Office, at Austin, Texas, as Second Class Mail Matter
Vol. VIII
MAY, 1893,
No. 11
DANIEL'S
MEDICAL
JOURNAL
F. E. DANIEL., M. D., Editor and Publisher, AUSTIN, TEXAS.
EUGENE VON BOECKMANN, PRINTER, AUSTIN, TEXAS.
				

